# Temperature explains intraspecific functional trait variation in *Phragmites australis* more effectively than soil properties

**DOI:** 10.3389/fpls.2023.1285588

**Published:** 2023-11-24

**Authors:** Zhichao Xu, Huamin Liu, Lu Wen, Jinghui Zhang, Xiaoyun Xin, Jinpeng Hu, Xin Kou, Dongwei Liu, Yi Zhuo, Lixin Wang

**Affiliations:** ^1^ School of Ecology and Environment, Inner Mongolia University, Hohhot, China; ^2^ Collaborative Innovation Center for Grassland Ecological Security (Jointly Supported by the Ministry of Education of China and Inner Mongolia Autonomous Region), Hohhot, China; ^3^ Ministry of Education Key Laboratory of Ecology and Resource Use of the Mongolian Plateau, Hohhot, China

**Keywords:** functional traits, intraspecific variation, lakeshore wetland, plant economics spectrum, *Phragmites australis*, spatial scales

## Abstract

Common reed (*Phragmites australis*) is a widespread grass species that exhibits a high degree of intraspecific variation for functional traits along environmental gradients. However, the mechanisms underlying intraspecific variation and adaptation strategies in response to environmental gradients on a regional scale remain poorly understood. In this study, we measured leaf, stem, and root traits of common reed in the lakeshore wetlands of the arid and semi-arid regions of the Inner Mongolia Plateau aiming to reveal the regional-scale variation for functional traits in this species, and the corresponding potentially influencing factors. Additionally, we aimed to reveal the ecological adaptation strategies of common reed in different regions using the plant economics spectrum (PES) theory. The results showed that functional-trait variation followed significant latitudinal and longitudinal patterns. Furthermore, we found that these variations are primarily driven by temperature-mediated climatic differences, such as aridity, induced by geographical distance. In contrast, soil properties and the combined effects of climate and soil had relatively minor effects on such properties. In the case of common reed, the PES theory applies to the functional traits at the organ, as well as at the whole-plant level, and different ecological adaptation strategies across arid and semi-arid regions were confirmed. The extent of utilization and assimilation of resources by this species in arid regions was a conservative one, whereas in semi-arid regions, an acquisition strategy prevailed. This study provides new insights into intraspecific variations for functional traits in common reed on a regional scale, the driving factors involved, and the ecological adaptation strategies used by the species. Moreover, it provided a theoretical foundation for wetland biodiversity conservation and ecological restoration.

## 1 Introduction

Plant functional traits are adaptive and effective traits that affect plant survival, growth, and reproduction; furthermore, they reflect plant responses to the environment (Liu and Ma, 2015; Bruelheide et al., 2018b) and have proved useful for predicting plant community assembly and diversity (Lavorel and Garnier, 2010), which can respond strongly to environmental and climate change, and serve as excellent ecological indicators (Joswig et al., 2022).

Numerous empirical studies have elucidated the nature of trait variation and have shown systematic relationships between traits and climatic and/or soil properties (Wright et al., 2005; Cornwell and Ackerly, 2009; Simpson et al., 2016). However, the dominant theories and methods of trait-based community ecology mainly focus on the differences in functional traits between species (Roscher et al., 2018), whereas the important role of intraspecific trait variation has been neglected (Siefert et al., 2015; Ma et al., 2022). Species with a widespread distribution tend to have greater trait variability and high intraspecific variation; moreover, they show a continuous geographic gradient, especially across different latitudes, as a consequence of acclimation to a broad range of environmental conditions (Siefert et al., 2015; Ren et al., 2020). Indeed, numerous studies have shown that large-scale variation for individual plant traits is associated with environmental gradients (Atkin et al., 2015; Wright et al., 2017). Further, previous studies have suggested that, together, climate and soil determine the form and function of plants (Simpson et al., 2016; Bruelheide et al., 2018a). However, studies on the characteristics of intraspecific variation for plant functional traits and their adaptation to environmental conditions are important for understanding plant community assembly and diversity in different regions, and the mechanisms underlying their response to habitat conditions (Suding et al., 2008; Kong et al., 2021).

The underlying concept of the PES theory has attracted wide attention from ecologists. Initially, Wright et al. (2004) defined the “leaf economic spectrum (LES),” as a continuously varying spectrum of leaf functional trait combinations at a global scale, to illustrate trade-off strategies between vascular plant resource acquisition and storage, which can be expressed through the range of variation in trait indicators and their quantitative relationships (Wright et al., 2004). However, since then, ecologists have extended LES studies to stem, root, whole plant, community structure, and ecosystem types at different levels (Pérez-Ramos et al., 2012; Kong et al., 2019; Li et al., 2019). One end of the PES represents the “fast investment-gain” strategy of plants, whereby, species make a low tissue investment and get a fast return, i.e., an acquisition strategy. Meanwhile, the other represents the “slow investment-gain” strategy of plants, whereby, species make a high tissue investment and gte a slow return, i.e., a conservative strategy (Wright et al., 2004; Reich and Cornelissen, 2014). PES studies can better describe the trade-offs between functional traits at the organ level. In particular, the PES theory provides new perspectives and explores avenues for researching intraspecific variation in functional traits and species ecological-adaptation strategies.

The cosmopolitan species, *P. australis*, is a tall wetland grass with high intraspecific variation that maks it a suitable model species for studying the mechanisms underlying intraspecific functional trait variation (Kueffer et al., 2013; Eller et al., 2017). In this study, we surveyed *P. australis* community sites in 13 lakeshore wetlands, and monitored 26 plant functional traits, including leaf, stem and root traits to analyze the variation for such traits according to geographic location, and the corresponding determinants. Furthermore, we investigated the extent to which the major dimensions underpinning the form variation of *P. australis* in lakeshore wetlands can be attributed to gradients in climate and soil conditions, and to what extent these factors can jointly or independently explain form-variation in *P. australis* in lakeshore wetlands. Finally, the ecological adaptation strategies under different regional characteristics are summarized using economic spectrum analysis. Specifically, we assessed the following hypotheses: (1) Intraspecific variation in *P. australis* functional traits exhibits significant latitudinal and longitudinal gradients that can be explained by the combined effects of climate and soil. (2) Economic spectrum theory applies to the functional traits of *P. australis* populations, and the ecological adaptation strategies of *P. australis* traits depend on regions.

## 2 Materials and methods

### 2.1 Study area, experiment design and sampling

This study was conducted on lakeshore wetland ecosystems along lakes in the semi-arid and arid regions of Inner Mongolia, northern China (**Figure S1**; **Table S1**). Thirteen sampling sites in the lakeshore wetlands of 11 lakes (three sampling sites in Hulun Lake of Xheke (XHK), Mniaob (MNAB) and Glada (GLD)) were investigated across a large geographic range (38.70° - 49.32° N, 101.27° - 117.71° E) in which two distinct climatic regions were identified, as per aridity index (AI), namely, semi-arid (AI ≥ 0.2) and arid (AI < 0.2) regions (Antonio and Robert, 2019). Four of 11 sampling localities laid in the semi-arid region (Hulun lake, Buir Lake (BEL), Zagustai lake (ZGST), and Hongjiannao lake (HJN)), and seven in the arid region (Chagannur lake (CGNE), Narin Lake (NLH), Tonggunaoer lake (TG), Bagadabusu lake (BG), Badain lake fresh water (BDD), Badain lake salt (BDX), and Juyan Lake (JYH)) (**Figure S1**). Communities of *P. australis*-dominant species were selected in lakeshore wetlands to measure functional traits.

Fresh samples were collected from 13 sampling sites in the selected lakeshore wetlands during July-August 2020. Five quadrats (1 m×1 m, 5 blocks) were randomly selected from each *P. australis* community. In each quadrat, 8-10 intact plants with uniform growth and without pests or diseases were selected, excavated together with their roots, and brought back to the laboratory for the separation of organs to study the functional traits of *P. australis*. Additionally, topsoil samples (0 – 30 cm depth) were collected from three randomly selected sample quadrats of the excavated *P. australis* specimens. Five soil cores were collected from each quadrat using a 7-cm diameter drill, mixed into one composite sample, and brought back to the laboratory to analyze the soil physical and chemical properties.

### 2.2 Functional traits measurements

Twenty-six traits of common reed plants were measured directly or indirectly. Plant height was measured in the field, and selected individuals were excavated together with their root systems. Then, roots, stems, and leaves of each plant were separated and individually scanned at a 300 dpi resolution (Epson Perfection V850 Pro scanner, Dell USA), such as to measure the leaf area and root diameter infield before drying at 80°C for 48 h. Biomass was measured to approximate 10^−3^ g, and the specific leaf area (SLA), leaf dry matter content (LDMC), and specific root length (SRL) were calculated after Freschet et al. (2021). Scanned leaf and root images were analyzed using Photoshop (http://www.ps.lhfei.cn/) and WinRHIZO (http://www.regentinstruments.com/assets/winrhizo_about.html), respectively. Leaf thickness (LTH), stem diameter (SD) and root diameter (RD) were measured using Vernier calipers. Stem density (SDE) was calculated from dry stem weight and volume measurements. Carbon and nitrogen concentrations of these samples [including leaf nitrogen content (LN), leaf carbon content (LC), stem nitrogen content (SN), stem carbon content (SC), root nitrogen content (RN) and root carbon content (RC)] were measured using an elemental analyzer (Elementar Vario EL III, Germany). In turn, total phosphorus (TP) concentration, including leaf phosphorus content (LP), stem phosphorus content (SP) and root phosphorus content (RP) was measured using the H_2_SO_4_-HClO_4_ fusion method. The stoichiometric ratios of the roots, stems and leaves (including leaf C:N, C:P, and N: P ratios; stem C:N, C:P, and N: P ratios; root C:N, C:P, and N:P ratios) were calculated based on their C, N and P contents (Wang et al., 2020).

### 2.3 Soil property measurements and climate data collection

Soil samples were sieved through a 2-mm mesh sieve to remove roots and small rocks, and the divided into two parts: one was naturally dried, while the other was stored at 4°C as fresh soil sample. Fresh soil sample (10 g) was used to measure ammonium concentrations (NH4) with 50 ml of 2 M KCl-extractable with a continuous flow spectrophotometer (FIAstar 5000, Foss Tecator, Denmark). Soil available phosphorus (AP) was extracted with 0.5M NaHCO_3_ (pH 8.5) and analyzed using the molybdenum blue-ascorbic acid method. Soil pH was measured in a 1:5 soil:water suspension and soil electrical conductivity (EC) was measured in a 1:2.5 soil:water suspension using a glass electrode. Soil total carbon (TC) and total nitrogen (TN) were measured using an elemental analyzer (Elementar Vario EL III, Germany).

As for climate factors, mean annual temperature (MAT), mean annual precipitation (MAP), annual potential evapotranspiration (PET), and aridity index (AI) were used in this study. MAT and MAP data with a resolution of 30 × 30 s were obtained from the WorldClim Global Climate Database using the geographic coordinates of each plot (http://www.worldclim.org). PET data with a resolution of 30 × 30 s were extracted from CGIAR-CSI (http://www.cgiar-csi.org). AI was calculated as follows: AI = MAP/PET (Antonio and Robert, 2019).

### 2.4 Statistical analysis

All data were analyzed using R version 4.1.1 (R Core Team, 2021). Quadrat-level data were obtained by averaging the trait values of all sampled individuals in the quadrat. To meet the assumption of normality in subsequent tests, all data were log-transformed before analysis. The functional traits of *P. australis* were analyzed using principal component analysis (PCA), and the PC1 axis with the greatest amount of variability was used to represent leaf, stem, and root traits. We performed simple linear regression analysis to investigate the relationships between leaf, stem, and root traits, with latitudinal and longitudinal gradients. This analysis was used to reveal intraspecific variations on a large scale. Ridge regression (Friedman et al., 2009) examines all environmental variables to explain intraspecific variation in functional traits and is a well-established linear regression method that is suitable for dealing with a large number of collinear predictors of climate and soil variables. We used hierarchical partitioning, i.e., ridge regression with hierarchical partitioning (Chevan and Sutherland, 1991) to define the extent to which the explained intraspecific variablity for each trait on the latitudinal gradient was due to independent and joint climate- and soil- variable effects. Additionally, redundancy analysis (RDA) was used to detect how climate and soil factors affect intraspecific variation for functional traits (R package ‘vegan’); subsequently, the explanation of climate and soil factors was confirmed by package ‘rdacca.hp’ (Lai et al., 2022). The significance of RDA was tested using random permutations (n = 999 for all analyses). PCA was designed to rank leaf, stem, root, and whole-plant traits of *P. australis*. A higher proportion of the variance was explained by the calculated PC1 and PC2, which were used as proxies for LES, stem economics spectrum (SES), root economics spectrum (RES), and whole-plant economics spectrum (WPES) to reveal ecological adaptation strategies. The relative contribution of each trait to PC1 and PC2 was estimated by correlation analysis between each trait index, and PC1 and PC2 scores. The F-test was used to analyzed the differences between PC1 and PC2 scores for leaf, stem, root and whole-plant traits in the arid and semi-arid regions.

## 3 Results

### 3.1 Intraspecific variation for traits along latitudinal and longitudinal gradienst

**Figure 1:**
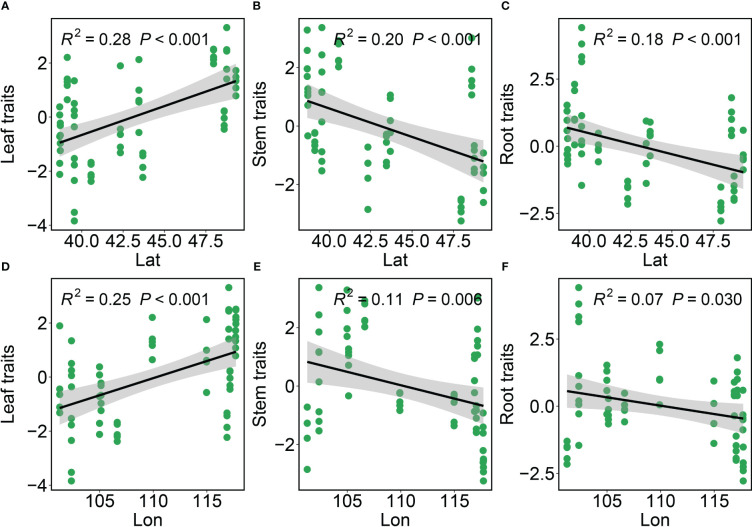
The intraspecific variation in the leaf traits, stem traits and root traits of *Phragmites australis* with latitudinal and longitudinal gradient. Black lines are the fitted lines from OLS regressions. Grey shadings represent 95% confidence intervals. Significance (*p*-value) is shown in parentheses. *R* ^2^ describes the proportion of variation explained by each model. Lat, Latitude; Lon, Longitude. **(A–C)** The variations of leaf, stem, and root traits along the latitude gradient. **(D–F)** The variations of leaf, stem, and root traits along the longitude gradient.

**Figure 2:**
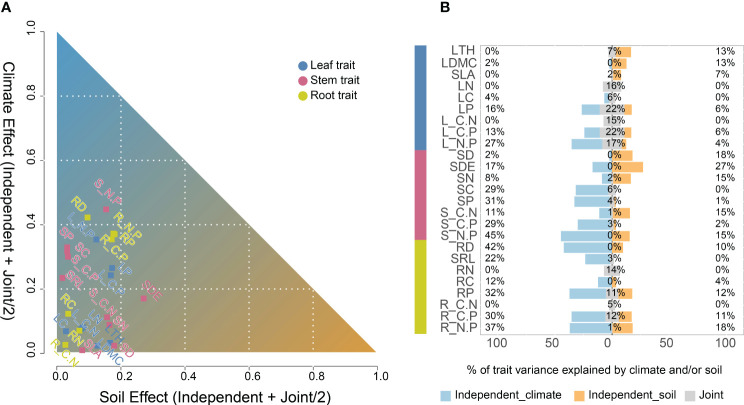
Hierarchical partitioning identifies the contribution of climate and soil variables to explained for each trait variation of *Phragmites australis* (n = 65; blue, leaf traits; red, stem traits; yellow, root traits). **(A)** The *x–y* plot of the soil versus climate factors to explained a trait variation. The axes showed the sum of the respective independent and joint effect of soil and climate by hierarchical partitioning. The independent effect was the fraction of *r^2^* explained exclusively by either soil or climate variables. The joint effect was the fraction explained by both climate and soil together, and was split equally among them. **(B)** Percentage variation explained by climate (purple, percentages on the left), soil (peach, percentages on the right) and jointly (grey, percentages in the middle) for trait groups-leaf, stem and root. Leaf thickness, LTH; Leaf dry matter content, LDMC; Specific leaf area, SLA; Leaf nitrogen content, LN; Leaf carbon content, LC; Leaf phosphorus content, LP; Leaf C/N ratio, L_C.N; Leaf C/P ratio, L_C.P; Leaf N/P ratio, L_N.P; Stem diameter, SD; Stem density, SDE; Stem nitrogen content, SN; Stem carbon content, SC; Stem phosphorus content, SP; Stem C/N ratio, S_C.N; Stem C/P ratio, S_C.P; Stem N/P ratio, S_N.P; Root diameter, RD; Specific root length, SRL; Root nitrogen content, RN; Root carbon content, RC; Root phosphorus content, RP; Root C/N ratio, R_C.N; Root C/P ratio, R_C.P; Root N/P ratio, R_N.P.

Leaf, stem and root functional traits in *Phragmites australis* showed a significant latitudinal and longitudinal gradient pattern (*P* ≤ 0.05), albeit minor to moderately explained (*R* ^2^ ≤ 0.28, **Figure 1**). Specifically, leaf trait values increased significantly (*R* ^2^ = 0.28, *P* < 0.001, **Figure 1A**; *R* ^2^ = 0.25, *P* < 0.001, **Figure 1D**) with increasing latitude and longitude, whereas stem and root trait values decreased significantly (*R* ^2^ < 0.23, *P* < 0.05, **Figures 1B, C, E, F**). The variation in functional traits of common reed along longitude and latitude was primarily attributed to changes in climate as reflected by the aridity index associated with the change in both longitude and latitude. We then assessed the independent or joint effects of climate and soil on intraspecific trait variation using ridge regression (**Figure 2**; **Table 1**). Root and stem traits (RR: ridge regression; *r* ^2^ *_stem_* = 0.60, *r* ^2^ *_root_* = 0.56) were better explained than leaf traits (*r* ^2^ *_leaf_* = 0.48, **Table 1**). Further, hierarchical partitioning (RR with hierarchical partitioning) results suggested that both independent and joint effects of climate and soil affected intraspecific trait variations (**Figure 2**). However, the independent climate effects were observed for most traits and explained trait variation better than any independent soil effect or joint climate-soil effects (**Figure 2B**; **Table 1**).

**Table 1:** The intraspecific variation in each trait of *Phragmites australis* explained (*r* ^2^) by ridge regression and the independent and joint effects for climate and soil variables from hierarchical partitioning.

Treat	Group	Explained variance by soil and climate (*r^2^*)	Independent_climate effect (*r^2^*)	Independent_soil effect (*r^2^*)	Joint effect (*r^2^*)
		Ridge regression model	Hierarchical partitioning	Hierarchical partitioning	Hierarchical partitioning
**LTH**	Leaf	0.20	0	0.07	0.13
**LDMC**	Leaf	0.15	0.02	0	0.13
**SLA**	Leaf	0.09	0	0.02	0.07
**LN**	Leaf	0.16	0	0.16	0
**LC**	Leaf	0.10	0.04	0.06	0
**LP**	Leaf	0.44	0.16	0.22	0.06
**L_C.N**	Leaf	0.15	0	0.15	0
**L_C.P**	Leaf	0.41	0.13	0.22	0.06
**L_N.P**	Leaf	0.48	0.27	0.17	0.04
**SD**	Stem	0.20	0.02	0	0.18
**SDE**	Stem	0.44	0.17	0	0.27
**SN**	Stem	0.25	0.08	0.02	0.15
**SC**	Stem	0.35	0.29	0.06	0
**SP**	Stem	0.36	0.31	0.04	0.01
**S_C.N**	Stem	0.27	0.11	0.01	0.15
**S_C.P**	Stem	0.34	0.29	0.03	0.02
**S_N.P**	Stem	0.60	0.45	0	0.15
**RD**	Root	0.52	0.42	0	0.10
**SRL**	Root	0.25	0.22	0.03	0
**RN**	Root	0.14	0	0.14	0
**RC**	Root	0.16	0.12	0	0.04
**RP**	Root	0.55	0.32	0.11	0.12
**R_C.N**	Root	0.05	0	0.05	0
**R_C.P**	Root	0.53	0.30	0.12	0.11
**R_N.P**	Root	0.56	0.37	0.01	0.18

Leaf thickness, LTH; Leaf dry matter content, LDMC; Specific leaf area, SLA; Leaf nitrogen content, LN; Leaf carbon content, LC; Leaf phosphorus content, LP; Leaf C/N ratio, L_C.N; Leaf C/P ratio, L_C.P; Leaf N/P ratio, L_N.P; Stem diameter, SD; Stem density, SDE; Stem nitrogen content, SN; Stem carbon content, SC; Stem phosphorus content, SP; Stem C/N ratio, S_C.N; Stem C/P ratio, S_C.P; Stem N/P ratio, S_N.P; Root diameter, RD; Specific root length, SRL; Root nitrogen content, RN; Root carbon content, RC; Root phosphorus content, RP; Root C/N ratio, R_C.N; Root C/P ratio, R_C.P; Root N/P ratio, R_N.P.

**Figure 3:**
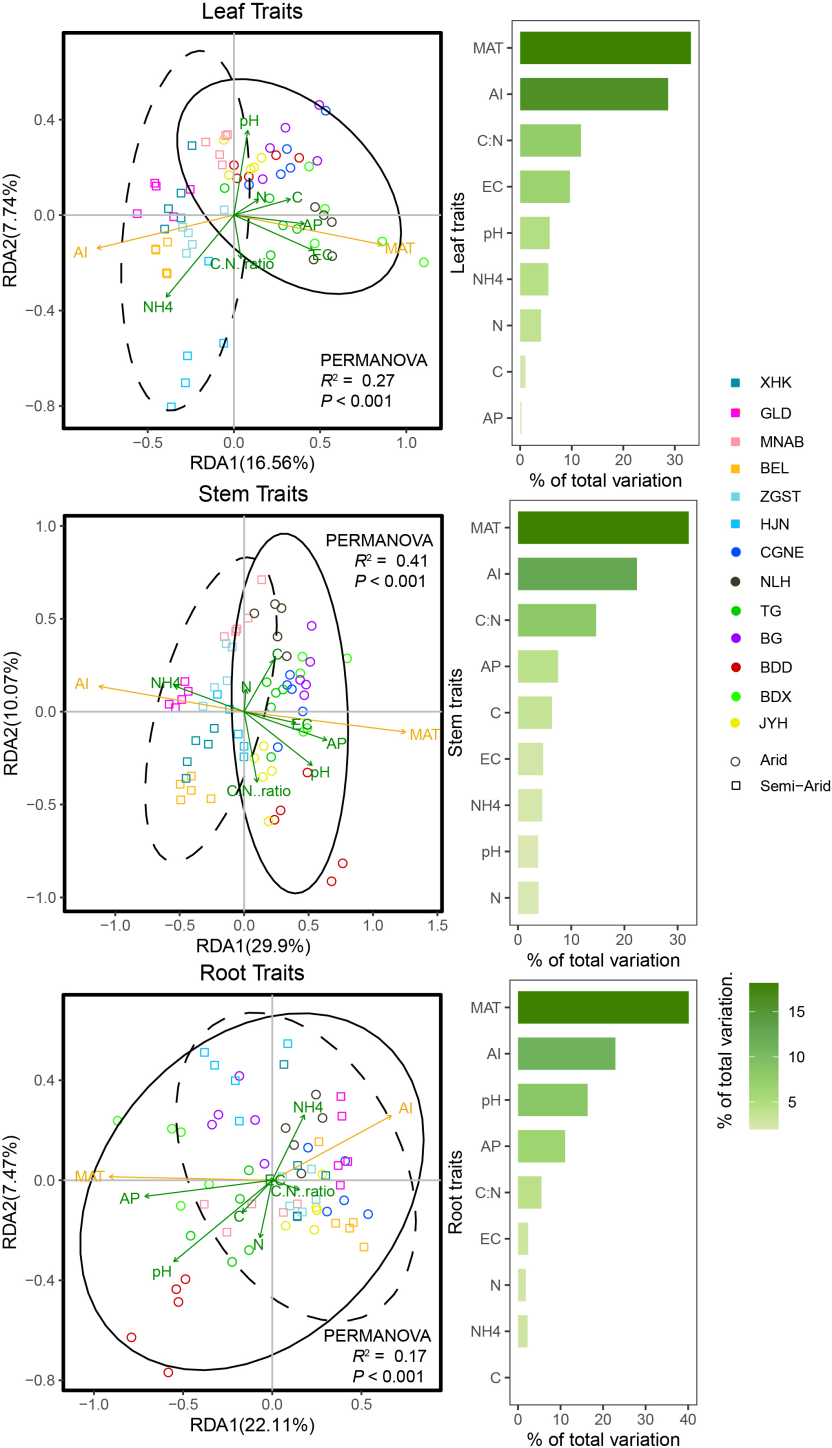
Redundancy analysis (RDA) ordination for leaf, stem, and root traits of *Phragmites australis* and contribution of climatic and soil factors to trait variability in lakeshore wetlands across arid and semi-arid regions (Permutation test (n = 999), *P* < 0.001). Arrows were colored according to climate and soil factors group (climate, yellow and soil, green variables; arrow length and point positions scaled to fit the plot). The solid lines represent 95% confidence intervals for functional traits in arid regions. Dashed lines represent 95% confidence intervals for functional traits in semi-arid regions. Bars plot each RDA plot show the variation explained by each factor.

Using RDA, we showed that climatic factors MAT and AI, and soil C:N, pH, and EC, were the best predictors of intraspecific trait variation. Furthermore, trait variation and the driving factors involved were significant in arid and semi-arid regions (**Figure 3**, *R* ^2^ > 0.17, *P* < 0.001). Specifically, MAT, pH, and EC were the best predictors of leaf-, stem-, and root-trait variation in arid regions, whereas AI and soil NH_4_ best predicted leaf-, stem-, and root-trait variation in semi-arid regions (**Figure 3**).

### 3.2 The economics spectrum of *P. australis* traits and ecological adaptation strategies

**Figure 4:**
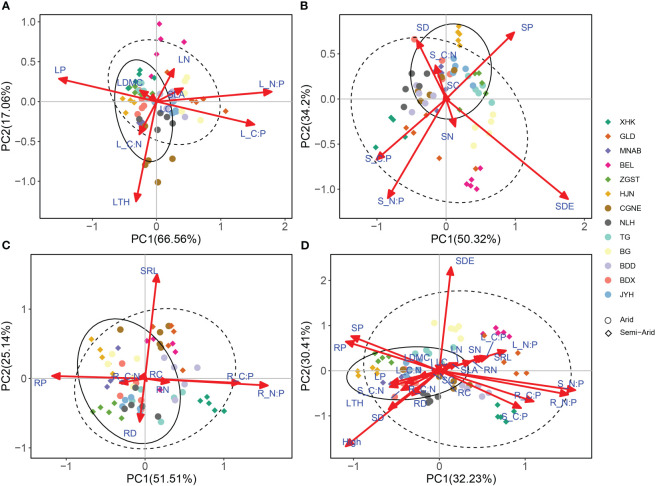
Principal component analysis (PCA) of leaf, stem, root system and whole-plant traits. **(A)** PCA of leaf traits. **(B)** PCA of stem traits. **(C)** PCA of root traits. **(D)** PCA of whole-plant traits. The solid lines represent 95% confidence intervals for functional traits in arid regions. Dashed lines represent 95% confidence intervals for functional traits in semi-arid regions. Leaf thickness, LTH; Leaf dry matter content, LDMC; Specific leaf area, SLA; Leaf nitrogen content, LN; Leaf carbon content, LC; Leaf phosphorus content, LP; Leaf C/N ratio, L_C.N; Leaf C/P ratio, L_C.P; Leaf N/P ratio, L_N.P; Stem diameter, SD; Stem density, SDE; Stem nitrogen content, SN; Stem carbon content, SC; Stem phosphorus content, SP; Stem C/N ratio, S_C.N; Stem C/P ratio, S_C.P; Stem N/P ratio, S_N.P; Root diameter, RD; Specific root length, SRL; Root nitrogen content, RN; Root carbon content, RC; Root phosphorus content, RP; Root C/N ratio, R_C.N; Root C/P ratio, R_C.P; Root N/P ratio, R_N.P.

Based on economics spectrum theory, the traits of *P. australis* showed an ecological adaptation strategy for conservation in arid regions, and one of acquisition in semi-arid regions (**Figure 4**, See Appendix S3 in Supporting Information). Particularly, the results of PCA for leaf trait variability showed that PC1 and PC2 explained 66.56% and 17.06% of the variance, respectively (**Figure 4A**). The PC1 axis represented a gradient from SLA and LN to LDMC and LTH, confirming a certain coordination among these leaf traits (**Figure 4A**). Most of the *P. australis* leaf traits in the arid region were clustered on the conservative side and those in the semi-arid region were clustered on the acquisitive side. The scores of the PC1 axis were significantly different between the arid and semi-arid regions (*p* < 0.001; **Table S3**).

In turn, the results of PCA for stem traits showed that PC1 and PC2 explained 50.32% and 34.2% of the variance, respectively (**Figure 4B**). The PC1 axis represented a gradient from SDE and SP to SD, confirming the coordination among these stem traits. Common reed specimens of arid and semi-arid regions were distributed on both the conserved and acquired sides but neither of them had any significant effect on the PC1 axis (*p* > 0.05, **Table S3**). Lastly, the results of PCA for root traits showed that PC1 and PC2 explained 51.51% and 25.14% of the variance, respectively, but there was no coordination in the traits along the PC1 axis (**Figure 4C**). Therefore, we did not detect an economics spectrum for *P. australis* roots in this study.

As for the results of PCA for whole-plant traits, this showed that PC1 and PC2 explained 32.23% and 30.41% of the variance, respectively (**Figure 4D**). Thus, whole-plant traits (except LDMC and RC) contributed significantly to the PC1 axis (**Table S2**), confirming the coordination of whole-plant traits along the PC1 axis. Most of the *P. australis* traits observed in semi-arid regions were distributed on the acquisition side, whereas as those in arid regions were distributed on the conservative side; furthermore, and the PC1 axis scores of the *P. australis* traits in the arid regions were significantly higher than those in the semi-arid regions (*p* < 0.001, **Table S3**).

## 4 Discussion

This study demonstrated that intraspecific trait variation in *P. australis* followed a pattern associated with a significant latitudinal and longitudinal gradient along the lakeshore wetland in Inner Mongolia. Furthermore, such variation basically depended on climatic differences as reflected on aridity index variability associated with longitude and latitude, and was less dependent on edaphic properties. Through PES analysis, we confirmed that the economics spectrum of *P. australis* plants not only existed in the traits of a single organ, but also acted at whole-plant level. Moreover, the traits of *P. australis* showed a conservative ecological adaptation strategy in arid regions and an acquisitively one in semi-arid regions.

### 4.1 Intraspecific functional-trait variation with location characteristics is regulated by climate

For widely distributed species, differences in stand and climatic environment in natural growth habitats inevitably lead to difference in plant physiological and ecological characteristics, resulting in significant differences in functional traits (Roscher et al., 2018). Thus, for example, previous studies have shown that specific leaf area and dry matter content are important indicators of plant resource utilization strategies (Joswig et al., 2022). In general, a lower specific leaf area, which restricts water vapor diffusion from the leaf out to the surrounding atmosphere, corresponds to thicker leaves (Dong et al., 2020). This adaptation enhances ability of the leaf to resist water loss, making it more effective under arid conditions (Wright et al., 2002; Rodriguez et al., 2015; Niinemets, 2016). Hence, plants adapted to harsh environments often have a high dry-matter content, which enhances stress tolerance and nutrients retention (Thomas et al., 2020). Thus, *P. australis* has adapted to high latitudes by reducing specific leaf area and increasing leaf dry-matter content, which is consistent with most studies on leaf functional traits (Luo et al., 2019; Gong et al., 2020).

As important elements for plant growth and development, C, N, and P contents and their stoichiometric characteristics not only reflect the ability of plants to produce assimilated products and their efficiency for nutrient utilization, but additionally, they determine the limiting factors controlling plant growth and development (Ordoñez et al., 2009; Zhang et al., 2020). Mineral element concentrations in plant tissues differ with the characteristics of geographical locations, most likely reflecting the effects of different nutrient requirements and assimilative capacities under different climatic conditions for growth (Heilmeier, 2019). In this study, leaf and root N content did not show a latitudinal gradient-associated pattern, which may have resulted from the unrestricted availability of N in the lakeshore wetlands of the geographic pattern. In turn, leaf, stem, and root P contents in *P. australis* plants increased along a decreasing latitudinal pattern, indicating that lakeshore wetlands in arid regions are more P-limited than those in semi-arid regions. Further, plant C:N ratio tended to increase as the latitudinal gradient, indicating that the C and N uptake and assimilation capacities of *P. australis* increased with higher latitudes (Wang et al., 2018). Additionally, both plant C:P and N:P ratios showed a decreasing trend as the latitudinal gradient, indicating that P was reduced by P limitation from arid to semi-arid lakeshore wetlands, and that the P supply was greater than those of C and N. Indeed, C:P and N:P ratios can be used to determine the soil availability of plant nutrients and are widely used to determine the limiting patterns of C, N, and P nutrients in plant-soil systems (Luo et al., 2017; Yang et al., 2018).

Up to 60% of intraspecific trait variation was explained by climate and soil variables in our study. Particularly, LTH, LDMC, SLA, LN, LP, SDI, and SRL, showed broad-scale correlations with climate and soil, and many of these traits showed geographic patterns (Wright et al., 2017). Moreover, temperature-dependent climatic factors were the dominant factors in the variation in the functional traits of *P. australis* at the regional scale, which were less related to soil properties (**Figure 2**). Specifically, ambient temperature, potential evapotranspiration, and precipitation have strong effects on plant growth, biomass, reproduction, and phenology (Cavender-Bares et al., 2016). This study reports latitudinal and longitudinal clines in leaf, stem, and root traits, proving that the functional traits under study were preserved and expressed according to their adaptation to climatic factors at the place of origin, especially MAT (Ren et al., 2020). Several previous studies on *P. australis* found that this variation tends to follow patterns associated with climate changes along latitudinal and longitudinal gradients (Lin et al., 2010; Bhattarai et al., 2017; Gong et al., 2020). High and frequent precipitation, abundance of water vapor, and suitable temperatures, should meet the basic requirements of a healthy and vigorous plant physiological performance (Joswig et al., 2022). In addition, these conditions promote the weathering of soil minerals, provide suitable conditions for microbial activity, contribute to the rapid turnover of organic matter, and provide sufficient nutrients to microorganisms. In short, they represent conditions that allow plants to grow fast and tall in the race for light (Slessarev et al., 2016; Wright et al., 2017).

### 4.2 Plant economics spectrum and ecological adaptation strategies

The study of the economics spectrum provides new theories and methods for analyzing the effects of global climate change on plants and their adaptation mechanisms, whereby it has become a hot topic in ecological research (Sakschewski et al., 2015). In accordance with the criteria outlined by Wright et al. (2004) for defining LES, the first principal axis of trait covariation, representing a core set of traits explained a substantial proportion of the variance for LES, SES, RES, and WPES. The results of this study on a single *P. australis* population showed that the economics spectrum theory was equally applicable to different traits of the same species in different habitats. Furthermore, PES studies have pointed out the characteristics of different plant functional trait combinations and the interrelationships among traits, indicating that plants of different habitats adopt different environmental-adaptation strategies through trade-offs among traits (Niinemets, 2015; Riva et al., 2016).

Economic spectrum theory was applied to leaf, stem and whole plant traits of *P. australis*, and significant differences for LES and WPES in arid and semi-arid regions were detected. Common reed specimens in the arid region were distributed on the conservative side represented by LES and WPES, with a small investment in SLA, LN, L_C:P and L_N:P, and a large one in LDMC, LP and L_C:N. In contrast, specimens in the semi-arid region were distributed on the acquisition side. This suggested that *P. australis* has constant or consistent conservative or acquisitive strategies at the organ and whole plant levels in arid and semi-arid regions, respectively (Joswig et al., 2022). Thus, in arid zones, *P. australis* usually has thicker leaves and stronger stalks, and allocates more biomass to mechanical support, thereby exhibiting a conservative strategy for withstanding adverse environmental conditions (Pérez-Ramos et al., 2012). However, in semi-arid zones, where greater water and nutrients availability provide superior conditions for the growth of *P. australis*, an acquisition-based strategy was favored to meet the higher nutrient content required. In the lakeshore wetlands of Inner Mongolia, LES, SES, and WPES of *P. australis* showed significant differences between arid and semi-arid regions, whereas RES did not. We speculate that this may be due to the significant variation in root traits, most of which show a high coefficient of variation and are more sensitive to soil texture and nutrient contents, and *P. australis* specimens can display different survival strategies depending on the region (Packer et al., 2017; Feng et al., 2020). Thus, despite the importance of the selection of plant traits under different environmental conditions, the coordination of plant acquisition or conservation strategies among traits, organs, and resources still converges under different habitat conditions (Reich and Cornelissen, 2014).

## 5 Conclusions

This study revealed that functional traits of *P. australis* followed patterns significantly associated with latitudinal and longitudinal gradients in lakes and lakeshore wetlands within the arid and semi-arid regions of the Inner Mongolian Plateau. The resulting intraspecific variation in traits across latitudinal and longitudinal gradients was primarily influenced by temperature-mediated climatic factors, whereas correlations between such trait variations and soil heterogeneity or the combined effects of climate and soil were low. The economics spectrum of *P. australis* populations in leaf and stem traits, and in the whole-plant, was clearly established. Further, the “investment-gain” strategy axis of the economics spectrum of *P. australis* in arid and semi-arid regions was characterized by divergence into two distinct directions. The arid region showed a conservative strategy, whereas the semi-arid region showed an acquisitive strategy. Our data strongly support the research of WPES of a single species with a plant economics spectrum, and further enrich the integration of intraspecific variation and the plant economics spectrum in different climatic regions. These findings bear important theoretical and practical significance for understanding the ecological adaptation strategies of plant species.

## Data availability statement

The raw data supporting the conclusions of this article will be made available by the authors, without undue reservation.

## Author contributions

ZX: Data curation, Formal Analysis, Investigation, Methodology, Software, Writing – original draft. HL: Conceptualization, Methodology, Writing – review & editing. LWe: Conceptualization, Funding acquisition, Methodology, Writing – review & editing. JZ: Formal Analysis, Methodology, Writing – review & editing. XX: Data curation, Investigation, Writing – original draft. JH: Data curation, Investigation, Writing – original draft. XK: Investigation, Writing – original draft. DL: Writing – review & editing. YZ: Writing – original draft. LWa: Conceptualization, Funding acquisition, Methodology, Writing – review & editing.
