# Supplementary material for: Temperature explains intraspecific functional trait variation in Phragmites australis more effectively than soil properties
Source: Front Plant Sci. 2023 Nov 24;14:1285588. doi: 10.3389/fpls.2023.1285588 (PMC10704352; doi:10.3389/fpls.2023.1285588)
Supplement: Supplementary file 1 [file DataSheet_1.docx]

# Supporting Information

**Article title:** Temperature explains intraspecific functional trait variation in Phragmites australis more effectively than soil properties

Zhichao Xu^1,2,3^, Huamin Liu^1,2,3^, Lu Wen^1,2,3^, Jinghui Zhang^1,2,3^, Xiaoyun Xin^1^, Jinpeng Hu^1^, Xin Kou^1^, Dongwei Liu^1,2,3^, Yi Zhuo^1,2,3^, Lixin Wang^1,2,3†^

^1^ College of Ecology and Environment, Inner Mongolia University, Hohhot 010021, China

^2^ Collaborative in novation Center for Grassland Ecological Security (Jointly Supported by the Ministry of Education of China and Inner Mongolia Autonomous Region), Hohhot 010021, China

^3^ Ministry of Education Key Laboratory of Ecology and Resource Use of the Mongolian Plateau, Hohhot 010021, China

^†^Corresponding author. E-mail address: Lxwang@imu.edu.cn (Lixin Wang)

Postal address: School of Ecology and Environment, Inner Mongolia University, No.235 College Road West, 010021 Hohhot, China.

**The following Supporting Information is available for this article:**

# Supporting Information Appendix S1

**Study Area and Background environment data**

**Figure S1** Geographical locations of lakes for each trait measured of *Phragmites australis* across lakeshore wetland that was used in this study. XHK, MNAB, and GLD, Located on the North, southwestern and southeastern shores of Hulun Lake, respectively in Hulunber; BEL, Buir Lake on China-Mongolia border; CGNE and ZGST, Chagannur lake and Zagustai lake in Xilingol; HJN, Hongjiannao lake in Ordos Inner Mongolia and Yulin in Shaanxi; NLH, Narin Lake in Bayanner; BG and TG, Bagadabusu lake and Tonggunaoer lake in Tenggeli desert of Alxa; BDD and BDX, Badain lake fresh water and Badain lake salt in Badain Jaran Desert of Alxa; JYH, Juyan Lake in ejina of Alxa. Among them, XHK, MNAB, GLD, BEL, ZGST and HJN belong to the semi-arid region, and CGNE, NLH, BG, TG, BDD, BDX and JYH are located in the arid region. AI, aridity index.
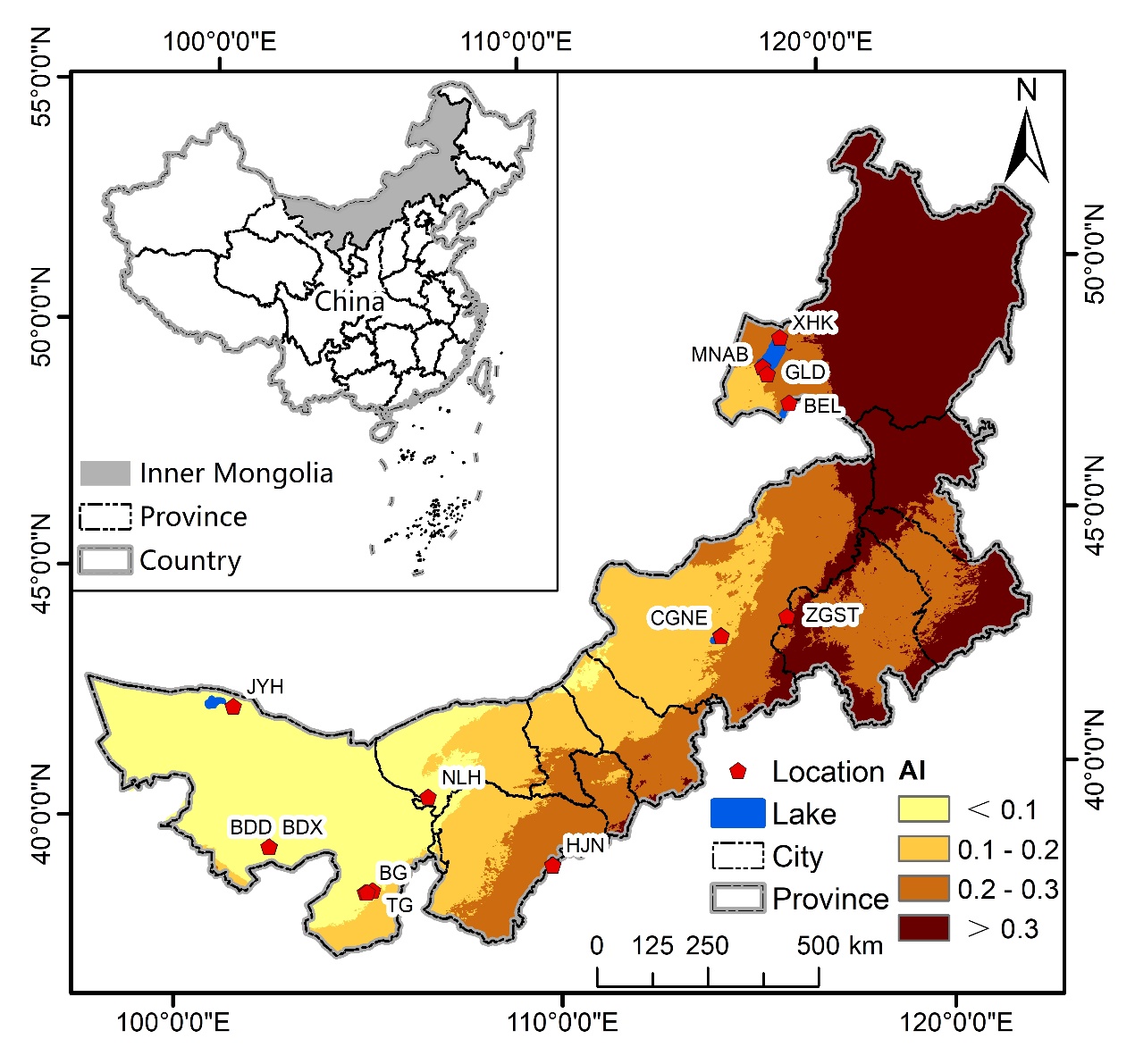


| Region | Semi-Arid | | | | | | Arid | | | | | | |
| --- | --- | --- | --- | --- | --- | --- | --- | --- | --- | --- | --- | --- | --- |
| Localities | XHK | GLD | MNAB | BEL | ZGST | HJN | CGNE | NLH | TG | BG | BDD | BDX | JYH |
| Lat (°N） | 49.32 | 48.76 | 48.61 | 47.96 | 43.69 | 39.12 | 43.45 | 40.56 | 38.70 | 38.67 | 39.55 | 39.55 | 42.33 |
| Lon (°E) | 117.68 | 117.05 | 117.16 | 117.71 | 116.88 | 109.91 | 114.97 | 106.64 | 105.13 | 104.94 | 102.37 | 102.36 | 101.27 |
| E (m) | 529.91 | 532.14 | 526.47 | 574.43 | 1289.51 | 1223.12 | 999.93 | 1038.01 | 1289.10 | 1270.01 | 1323.32 | 1318.06 | 899.50 |
| MAT (℃） | 0.28 | 0.78 | 0.89 | 0.48 | 0.93 | 7.05 | 2.82 | 8.19 | 8.99 | 9.11 | 9.06 | 9.16 | 8.34 |
| MAP (mm) | 295 | 247 | 255 | 264 | 337 | 370 | 244 | 118 | 158 | 155 | 88 | 90 | 43 |
| PET (mm yr^-1^) | 1052 | 1055 | 1142 | 1080 | 1148 | 1421 | 1283 | 1738 | 1727 | 1710 | 1860 | 1858 | 1823 |
| AI | 0.28 | 0.23 | 0.22 | 0.24 | 0.29 | 0.26 | 0.19 | 0.07 | 0.09 | 0.09 | 0.05 | 0.05 | 0.02 |

Table S1 Environmental features of the study localities of lakeshore wetlands in two regions with semi-arid and arid. Lat, latitude; Lon, longitude; E, elevation; MAT, mean annual temperature; MAP, mean annual precipitation; PET, potential evapo-transpiration; AI, aridity index. Semi-arid: 0.2 < AI < 0.5, Arid: AI < 0.2.

# Supporting Information Appendix S2

**Variation of leaf traits, stem traits and root traits of *Phragmites australis* in latitudinal gradient**

**Figure S2** The mean annual temperature (MAT) and aridity index (AI) varies with the latitudinal and longitudinal gradient.


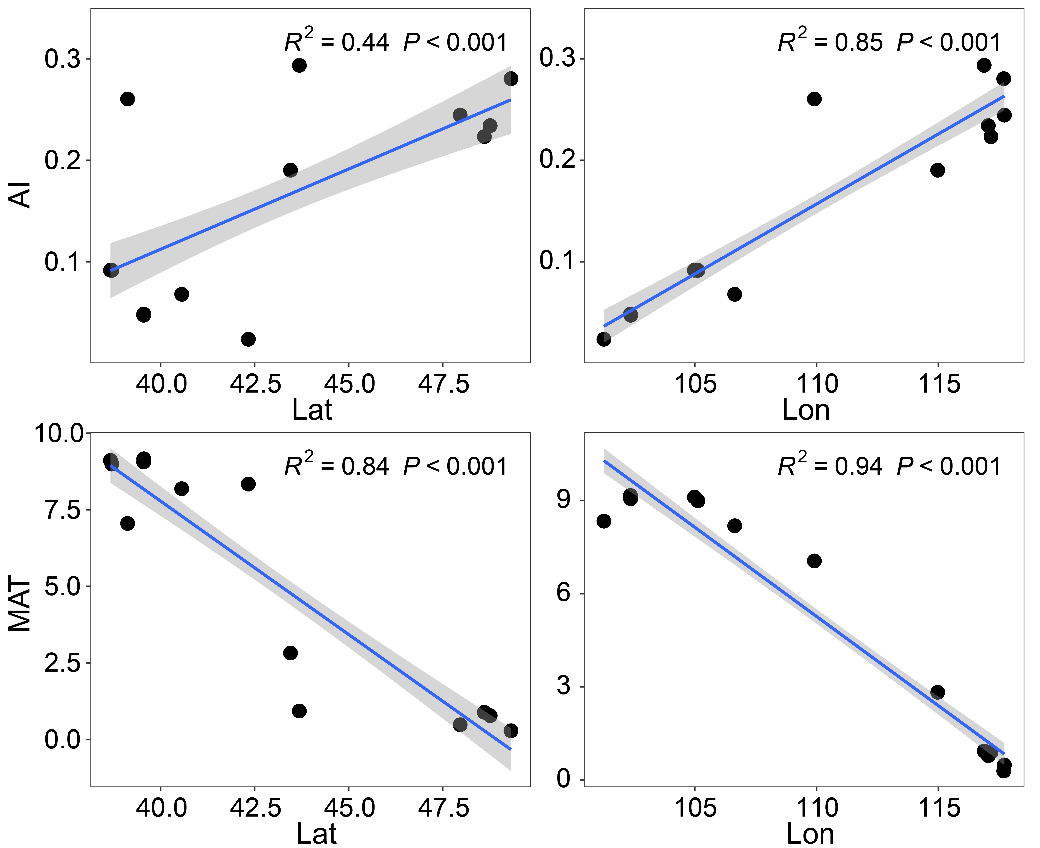


In the research, it was observed that the functional traits of *Phragmites australis* exhibit consistent trends along latitude and longitude gradients. Here, we will only present the latitudinal pattern.

**Figure S3** The intraspecific variation in the leaf traits of *Phragmites australis* with latitudinal gradient.


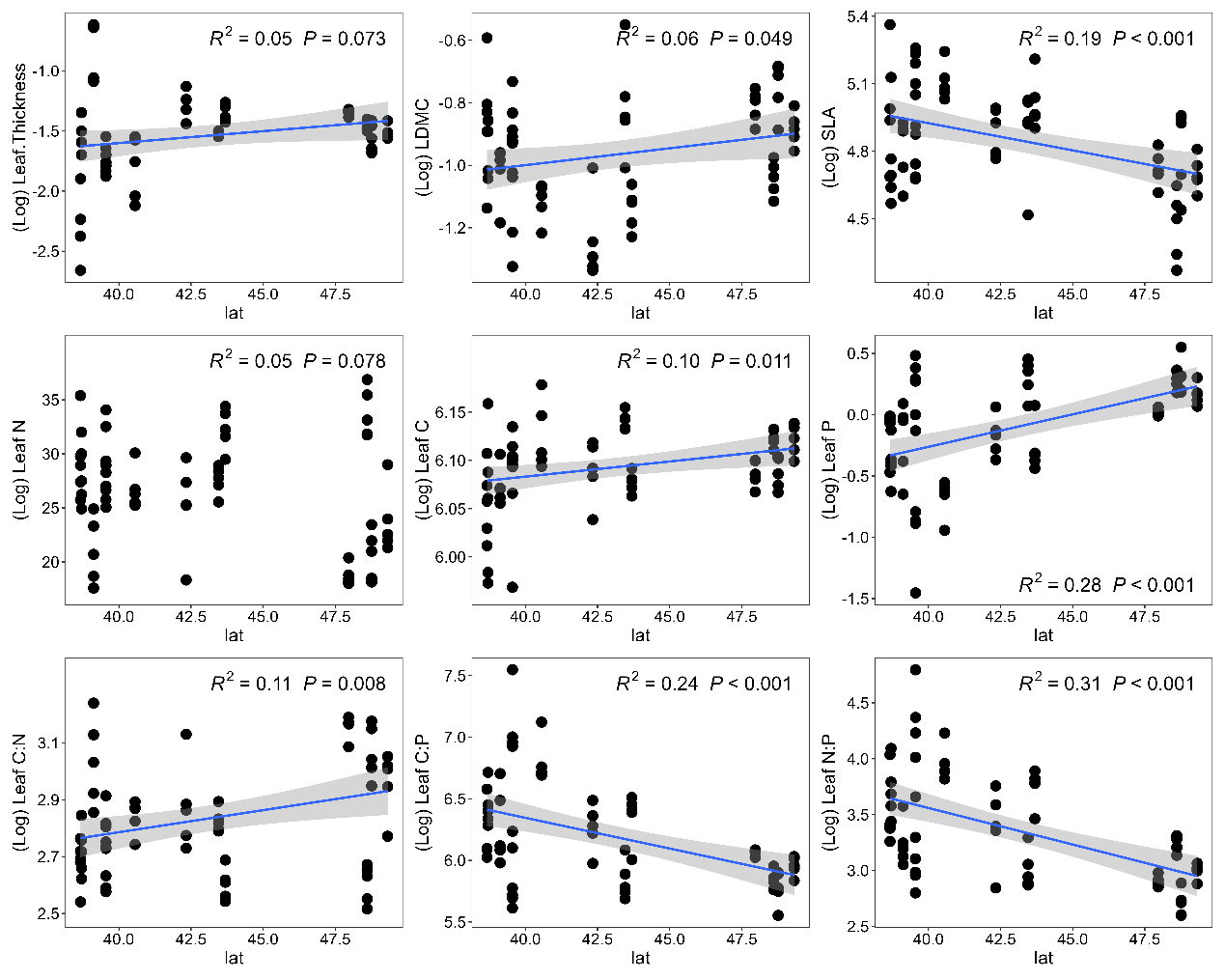


**Figure S4** The intraspecific variation in the stem traits of *Phragmites australis* with latitude gradient.


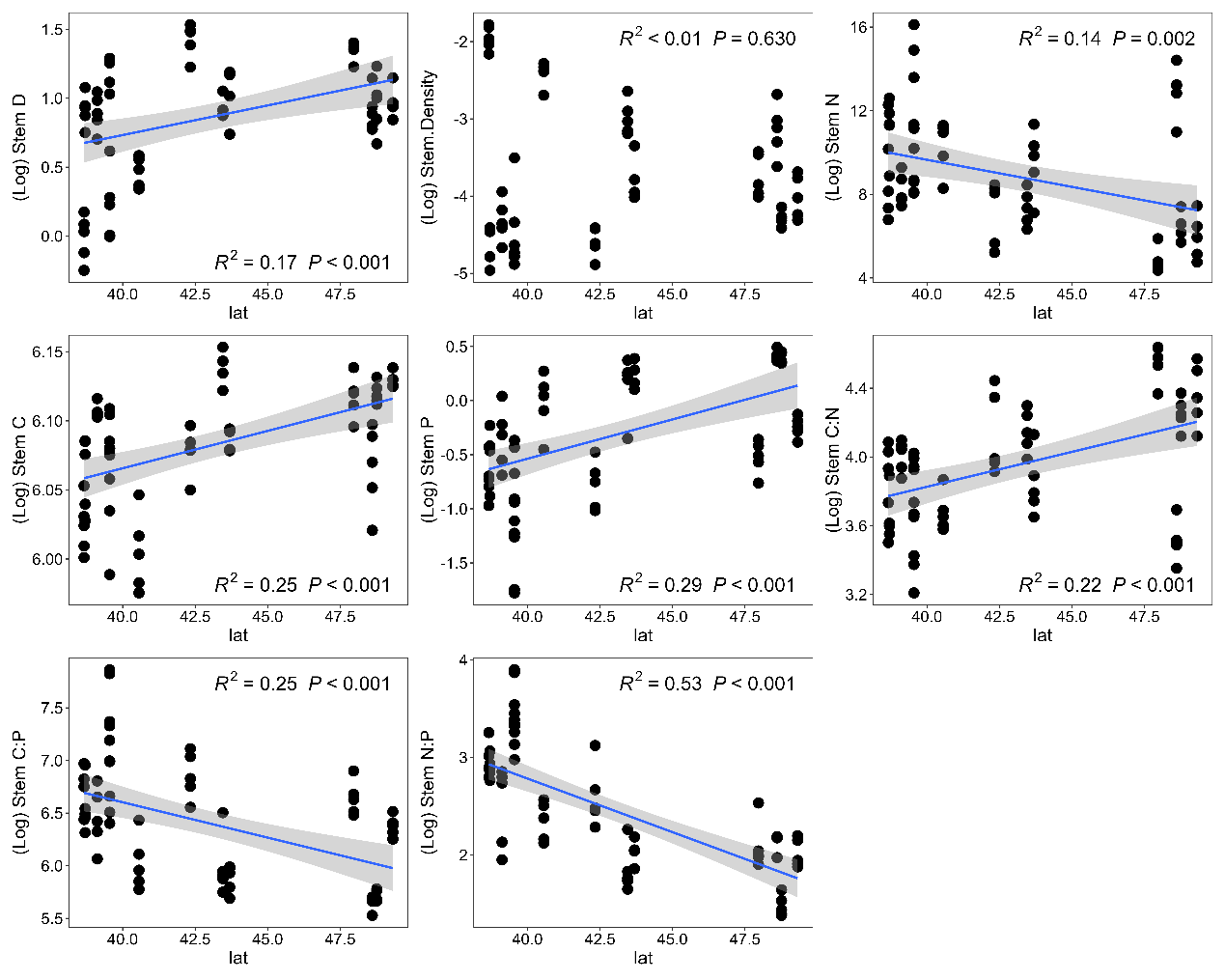


**Figure S5** The intraspecific variation in the root traits of *Phragmites australis* with latitude gradient.


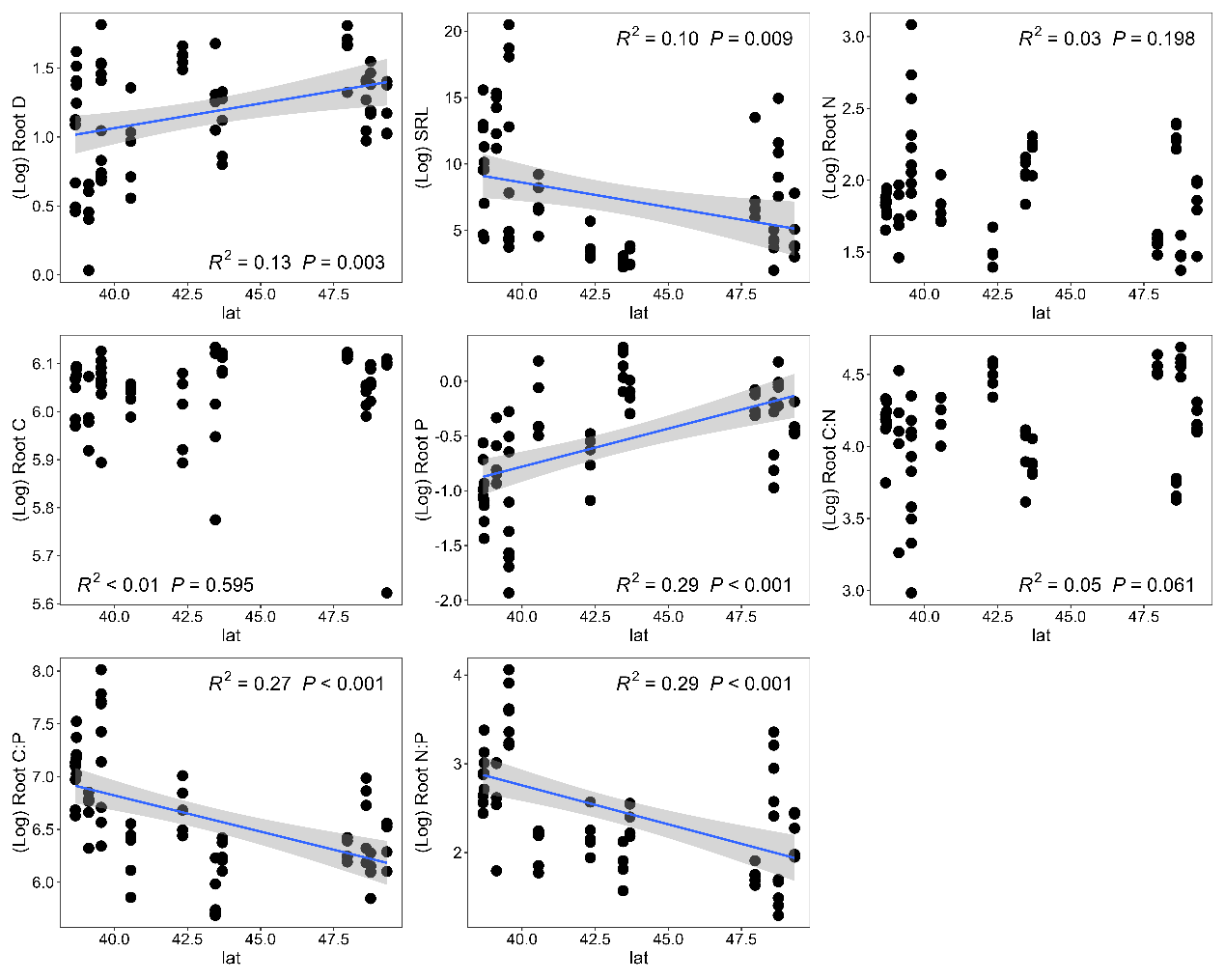


# Supporting Information Appendix S3

**Ecological adaptation strategies based on plant economic spectrum**

Based on the principal component analysis (PCA) (Fig. 4), revealed the resource utilization and ecological adaptation strategies of *P. australis* in different regions. The results of PCA for leaf traits showed that PC1 and PC2 occupied 66.56% and 17.06% of the variance explained, respectively (Fig. 4a). Except for LTH and LC, the remaining leaf traits contributed significantly to the PC1 axis (Table S2). One side of the PC1 axis exhibits *P. australis* plants with extensive SLA, LN, L_C:P and L_N:P, small LDMC, LP and L_C:N, showing a "fast investment-gain" (acquisition type) strategy. The other side has a small SLA, LN, L_C:P and L_N:P, and large LDMC, LP and L_C:N, exhibiting a "slow-investment-gain" (conservative) strategy. The PC1 axis is comparable to the "investment-return" strategy axis of the economic spectrum theory, which leads us to define an leaf economic spectrum (LES). Most of the *P. australis* in the arid region are clustered on the conservative side of the LES and the semi-arid region is clustered on the acquisitive side, and the scores of the PC1 axis were significantly different in the arid and semi-arid regions (*p* < 0.001, Table S3).

The results of PCA for stem traits showed that PC1 and PC2 occupied 50.32% and 34.2% of the variance explained, respectively (Fig. 4b). The SN, SC and S_C:N were not correlated with the PC1 axis, and all other stem traits contributed to the PC1 axis (Table S2). One side of the PC1 axis exhibits *P. australis* stem traits with large SDE, SN and SP, small SD, S_C:N, S_C:P and S_N:P, and a “fast investment-gain” type (acquisition type) strategy. The other side of the PC1 axis is characterized by small SDE, SN and SP, large SD, S_C:N, S_C:P and S_N:P, “slow investment-return” (conservative) strategies. The PC1 axis of the stem trait also corresponds to the “investment-return” strategy axis in the economic spectrum and can define a stem economic spectrum (SES). Arid and semi-arid region *P. australis* were distributed on both conserved and acquired sides of the SES representation, and both were not significant on the PC1 axis (*p* > 0.05, Table S3).

The results of PCA for root traits showed that PC1 and PC2 occupied 51.51% and 25.14% of the variance explained, respectively (Fig. 4c). However, there was no coordination between root traits along the PC1 axis. Only RP, R_C:N, R_C:P and R_N:P contributed with the PC1 axis of root traits, and RD and SRL with the PC2 axis, so the economic spectrum of *P. australis* roots was not present (Table S2).

The results of PCA for whole-plant traits showed that PC1 and PC2 occupied 32.23% and 30.41% of the variance explained, respectively (Fig. 4d). All traits except LDMC and RC contributed significantly to the PC1 axis (Table S2). One side of the PC1 axis exhibited *P. australis* traits with extensive SLA, SRL, SDE, LN, SN, RN, L_C:P, L_N:P, S_C:P, S_N:P, R_C:P, R_N:P, and small SD, RD, LTH, LP, SP, RP, L_C:N, S_C:N, R_C:N, exhibiting "fast investment-income" (acquisition) strategy. The PC1 axis can be defined as the "investment-return" strategy axis in the whole-plant economic spectrum theory (WPES). Most of the *P. australis* in semi-arid regions were distributed on the acquisition side and those in arid regions on the conservative side, and the PC1 axis scores for *P. australis* traits were significantly different in the arid and semi-arid regions (*p* < 0.001, Table S3).

Table S2 Correlation coefficients between individual traits and the scores of the first and second principal components in each of the leaf, stem, root and whole-plant economics spectrum. (*, *P* < 0.05; **, *P* < 0.01; ***, *P* < 0.001).

| Traits |  | Leaf | | | | Stem | | | | Root | | | | Whole-Plant | | | | |
| --- | --- | --- | --- | --- | --- | --- | --- | --- | --- | --- | --- | --- | --- | --- | --- | --- | --- | --- |
|  |  | PC1 |  | PC2 |  | PC1 |  | PC2 |  | PC1 |  | PC2 |  | PC1 |  | PC2 |  |  |
| Leaf | LTH | -0.185 |  | -0.879 | ^***^ |  |  |  |  |  |  |  |  | -0.325 | ^**^ | -0.335 | ^**^ |  |
|  | LDMC | -0.361 | ^**^ | 0.207 |  |  |  |  |  |  |  |  |  | -0.176 |  | 0.021 |  |  |
|  | SLA | 0.481 | ^***^ | 0.234 |  |  |  |  |  |  |  |  |  | 0.247 | ^*^ | 0.279 | ^*^ |  |
|  | LN | 0.349 | ^**^ | 0.524 | ^***^ |  |  |  |  |  |  |  |  | 0.367 | ^**^ | 0.320 | ^**^ |  |
|  | LC | 0.025 |  | -0.011 |  |  |  |  |  |  |  |  |  | -0.272 | ^*^ | 0.272 | ^*^ |  |
|  | LP | -0.934 | ^***^ | 0.204 |  |  |  |  |  |  |  |  |  | -0.465 | ^***^ | -0.232 |  |  |
|  | L_C:N | -0.368 | ^**^ | -0.569 | ^***^ |  |  |  |  |  |  |  |  | -0.457 | ^***^ | -0.257 | ^*^ |  |
|  | L_C:P | 0.926 | ^***^ | -0.147 |  |  |  |  |  |  |  |  |  | 0.471 | ^***^ | 0.240 |  |  |
|  | L_N:P | 0.949 | ^***^ | 0.032 |  |  |  |  |  |  |  |  |  | 0.541 | ^***^ | 0.285 | ^*^ |  |
| Stem | SDI |  |  |  |  | -0.474 | ^***^ | 0.566 | ^***^ |  |  |  |  | -0.437 | ^***^ | -0.725 | ^***^ |  |
|  | SDE |  |  |  |  | 0.625 | ^***^ | -0.633 | ^***^ |  |  |  |  | 0.247 | ^*^ | 0.791 | ^***^ |  |
|  | SN |  |  |  |  | 0.185 |  | -0.392 | ^**^ |  |  |  |  | 0.544 | ^***^ | 0.340 | ^**^ |  |
|  | SC |  |  |  |  | -0.211 |  | 0.653 | ^***^ |  |  |  |  | -0.626 | ^***^ | -0.499 | ^***^ |  |
|  | SP |  |  |  |  | 0.721 | ^***^ | 0.564 | ^***^ |  |  |  |  | -0.639 | ^***^ | 0.432 | ^***^ |  |
|  | S_C:N |  |  |  |  | -0.171 |  | 0.461 | ^***^ |  |  |  |  | -0.594 | ^***^ | -0.344 | ^**^ |  |
|  | S_C:P |  |  |  |  | -0.766 | ^***^ | -0.484 | ^***^ |  |  |  |  | 0.585 | ^***^ | -0.565 | ^***^ |  |
|  | S_N:P |  |  |  |  | -0.635 | ^***^ | -0.676 | ^***^ |  |  |  |  | 0.832 | ^***^ | -0.341 | ^**^ |  |
| Root | RD |  |  |  |  |  |  |  |  | -0.046 |  | -0.593 | ^***^ | -0.265 | ^*^ | -0.543 | ^***^ |  |
|  | SRL |  |  |  |  |  |  |  |  | 0.088 |  | 0.961 | ^***^ | 0.316 | ^*^ | 0.214 |  |  |
|  | RN |  |  |  |  |  |  |  |  | 0.312 |  | 0.032 |  | 0.366 | ^**^ | 0.193 |  |  |
|  | RC |  |  |  |  |  |  |  |  | 0.096 |  | -0.189 |  | -0.011 |  | -0.207 |  |  |
|  | RP |  |  |  |  |  |  |  |  | -0.901 | ^***^ | -0.042 |  | -0.761 | ^***^ | 0.387 | ^**^ |  |
|  | R_C:N |  |  |  |  |  |  |  |  | -0.401 | ^***^ | 0.012 |  | -0.422 | ^***^ | -0.282 | ^*^ |  |
|  | R_C:P |  |  |  |  |  |  |  |  | 0.884 | ^***^ | -0.151 |  | 0.666 | ^***^ | -0.526 | ^***^ |  |
|  | R_N:P |  |  |  |  |  |  |  |  | 0.913 | ^***^ | -0.073 |  | 0.738 | ^***^ | -0.381 | ^**^ |  |

Table S3 Differences in PC1 and PC2 scores between semi-arid and arid regions (mean ± SE)

| PCA | Axis | Region | | Significant test | |
| --- | --- | --- | --- | --- | --- |
|  |  | Semi-Arid | Arid | *F-*value | *P* |
| Leaf | PC1 | -0.16±0.04 | 0.13±0.07 | 14.03 | 0 |
|  | PC2 | -0.16±0.06 | 0.14±0.06 | 15.86 | 0 |
| Stem | PC1 | 0.08±0.05 | -0.07±0.09 | 1.88 | 0.176 |
|  | PC2 | 0.30±0.04 | -0.26±0.07 | 46.45 | 0 |
| Root | PC1 | -0.16±0.05 | 0.14±0.08 | 10.16 | 0.002 |
|  | PC2 | 0.02±0.07 | -0.01±0.08 | 0.09 | 0.759 |
| Whole-plant | PC1 | -0.34±0.06 | 0.29±0.09 | 33.68 | 0 |
|  | PC2 | -0.05±0.04 | 0.04±0.13 | 0.45 | 0.505 |
